# The Combined Use of Melatonin and an Indoleamine 2,3-Dioxygenase-1 Inhibitor Enhances Vaccine-Induced Protective Cellular Immunity to HPV16-Associated Tumors

**DOI:** 10.3389/fimmu.2018.01914

**Published:** 2018-08-22

**Authors:** Ana C. R. Moreno, Bruna F. M. M. Porchia, Roberta L. Pagni, Patrícia da Cruz Souza, Rafael Pegoraro, Karine B. Rodrigues, Tácita B. Barros, Luana R. de Melo Moraes Aps, Eliseu F. de Araújo, Vera L. G. Calich, Luís C. de Souza Ferreira

**Affiliations:** ^1^Vaccine Development Laboratory, Department of Microbiology, Biomedical Sciences Institute, University of São Paulo, São Paulo, Brazil; ^2^Department of Clinical Chemistry and Toxicology, Faculty of Pharmaceutical Sciences, University of São Paulo, São Paulo, Brazil; ^3^Department of Immunology, Biomedical Sciences Institute, University of São Paulo, São Paulo, Brazil

**Keywords:** melatonin, 1-methyl-tryptophan, indoleamine 2, 3 dioxygenase, human papillomavirus, cancer immunotherapy

## Abstract

Immunotherapy has become an important ally in the fight against distinct types of cancer. However, the metabolic plasticity of the tumor environment frequently influences the efficacy of therapeutic procedures, including those based on immunological tools. In this scenario, immunometabolic adjuvants arise as an alternative toward the development of more efficient cancer therapies. Here we demonstrated that the combination of melatonin, a neuroimmunomodulator molecule, and an indoleamine 2,3-dioxygenase (IDO) inhibitor (1-methyl-DL-tryptophan, DL-1MT) improves the efficacy of an immunotherapy (gDE7) targeting human papillomavirus (HPV)-associated tumors. Melatonin or IDO inhibitors (D-1MT and DL-1MT) directly reduced proliferation, migration, adhesion and viability of a tumor cell line (TC-1), capable to express the HPV-16 E6 and E7 oncoproteins, but could not confer *in vivo* antitumor protection effects. Nonetheless, combination of gDE7 with melatonin or D-1MT or DL-1MT enhanced the antitumor protective immunity of gDE7-based vaccine in mice. Notably, expression of IDO1 in stromal cells and/or immune cells, but not in tumor cells, inhibited the antitumor effects of the gDE7, as demonstrated in IDO1-deficient mice. Finally, co-administration of gDE7, melatonin and DL-1MT further improved the protective antitumor effects and the numbers of circulating E7-specific CD8^+^ T cells in mice previously transplanted with TC-1 cells. The unprecedented combination of melatonin and IDO inhibitors, as immunometabolic adjuvants, thus, represents a new and promising alternative for improving the efficacy of immunotherapeutic treatments of HPV-associated tumors.

## Introduction

Human papillomaviruses (HPV) are widely spread pathogens responsible for one of the most common sexually transmitted diseases worldwide (1). Since the role in genital malignancies is well established, HPV, particularly the genotypes associated with tumor onset, is considered a relevant public health concern, causing approximately half a million deaths worldwide every year (1). Virtually all cervical cancers and around 90% of squamous anal cancers can be attributable to HPV infection (2). Furthermore, the correlation between HPV infection and other anogenital and oropharyngeal cancers is steadily growing (3). HPVs comprise a diverse group, and more than 200 HPV genotypes has been identified. The classification into low-risk or high-risk HPV genotypes relies on the oncogenic potential during persistent infection in the cervical tissue (2). In this scenario, the constitutive expression of E6 and E7 oncoproteins, leading to cellular transformation and immortalization, is mandatory for the onset and maintenance of HPV-associated cancers by high-risk genotypes (4). HPV-16 infection is more prevalent than any other high-risk HPV genotype in most regions worldwide (5).

HPV vaccination could considerably reduce the morbidity and mortality of cancers causally associated with this virus. However, after 12 years, many populations worldwide have not been vaccinated (6). Unfortunately, in several countries, HPV immunization rates are significantly lower than rates of other childhood and adolescents immunizations (7). Furthermore, cervical cancer remains as one of the most frequent causes of cancer-related deaths among women throughout the world (1), and current treatment approaches vary according to the clinical stage of the disease (8). Regarding metastatic/recurrent cervical cancer, chemotherapy is considered the first-line approach (9). However, the performance of chemotherapy, as well as other therapeutic interventions, drops dramatically in more advanced tumors, a direct consequence of the establishment of an immunosuppressive milieu in the tumor microenvironment and peripheral systems marked by accumulation of inhibitory cytokines and dysfunctional immune cells (10). In this scenario, the development of efficient immunotherapies or adjuvants, that ameliorate the immune suppressive environment created by tumor cells and improve the performance of conventional treatments, represents a priority and a necessity (11).

In recent decades, several studies documented that melatonin, a natural antioxidant molecule largely distributed among living organisms, plays a fundamental role in neuroimmunomodulation (12). In addition to the regulation of the circadian rhythms (13), melatonin also affects a diversity of physiological processes including immune functions. More precisely, melatonin significantly enhances the differentiation of type 1 helper T cells (Th1) and IFN-γ production (12), important steps for the activation of tumor-specific CD8^+^cytotoxic T cells. Additionally, melatonin has shown to have oncostatic and pro-apoptotic properties in a plethora of experimental tumor models and in different human tumor cell lines (14–16). Consequently, several studies have classified melatonin as a promising anticancer agent, including for combined therapies, with exciting potential to override the immunosuppressive environment associated with growing tumors. Concerning the challenge to overcome the tumor-mediated immunosuppression, melatonin showed inhibitory effects on the immunomodulatory enzyme indoleamine 2,3-dioxygenase-1 (IDO1) (16). Melatonin downregulates IDO1 at mRNA levels, as well as kynurenine production in skin cells and human melanoma cells (16). Moreover, melatonin synthesis can be stimulated by the racemic compound 1-methyl-DL-tryptophan (DL-1MT)(16), an inhibitor of IDO1 (17) and IDO2 (18).

It is well established that IDO1 expression suppresses innate and adaptive immune responses that, under certain circumstance, promote a tolerogenic microenvironment (19, 20). IDO1 acts in tryptophan degradation and kynurenine production that negatively regulates immune cells, leading to enhanced numbers of regulatory T cells (Treg) and myeloid-derived suppressor cells (MDSCs) (20). A key issue about cancer immune escape mechanisms lies in the ability of tumor cells to edit their phenotype using extrinsic tumor suppressor mechanisms. Sustained by this phenomenon, immunotherapy raised as a significant therapeutic breakthrough against tumor induced immune suppression (21). Since IDO1 is an endogenous mechanism of immune tolerance *in vivo*, IDO1 inhibitors are emerging as experimental molecules in oncology (22). Indeed, small-molecules inhibitors of IDO1, like epacadostat, navoximod and indoximod (D-1MT enantiomer), are under phase II or II/III clinical trials (23, 24). However, based on recent negative results of ECHO-301/KEYNOTE-252 phase 3 trial in metastatic melanoma (clinical trial information: NCT02752074) (25), many trials, particularly those that use the IDO inhibitor epacadostat, needed to be halted. Still, researchers have shown that different therapeutic combinations may subvert the failure of clinical trials, pointing that IDO inhibitors should not be abandoned for cancer immunotherapy (clinical trial information: NCT01961115, NCT02077881) (26, 27).

In the present study, we evaluated the therapeutic potential of a novel immunotherapy focusing on three components: melatonin, 1MT and an HPV-16 therapeutic vaccine (gDE7) based on a recombinant protein generated after the genetic fusion of the HPV-16 E7-oncoprotein with the envelope glycoprotein (gD) of herpes virus simplex virus (HSV). The IDO inhibitors 1MT (enantiomers and racemic mixture) were chosen to compose our therapeutic approach based on their preclinical and clinical data as adjuvants of antitumor therapies (20, 22, 23, 27). The gDE7 vaccine (28, 29), as well as its DNA version (pgDE7h) (30), has shown excellent therapeutic effects in experimental conditions based in mice transplanted with TC-1 cells, a murine tumor cell line encoding the HPV-16 oncoproteins. We show here that IDO1 expression in immune cells and stromal cells, but not in tumor cells, impairs the antitumor effect of the gDE7 vaccine and, more relevantly, we demonstrated that combination of melatonin and an IDO inhibitor augmented the antitumor therapeutic effects of gDE7 and increased the activation of E7-specific cytotoxic CD8^+^ T-cell responses. Our findings highlight the role of IDO1 as an important immunosuppression inducer that may impair the proper functioning of immunotherapy. Furthermore, we propose the unprecedented association of melatonin and IDO inhibitors as immunometabolic adjuvants for cancer immunotherapy.

## Materials and methods

### Cell culture

The TC-1 tumor cell line (31) was kindly provided in 2002 by Dr. T.C. Wu from Johns Hopkins University in Baltimore, MD, USA. The cells were cultured as previously described (29).

### IDO1 flow cytometer analysis

TC-1 cells were cultured until reach 90% of confluence, and then were harvested with trypsin and seeded in 96-well plates at a concentration of 5 × 10^5^ cell/well. Cells were washed with MACS buffer [phosphate-buffered saline (PBS), pH 7.2, 0.5% bovine serum albumin (BSA), and 2 mM EDTA], fixed and permeabilized with the BD Cytofix/Cytoperm™ Plus Buffer Set (#555028,) and intracellularly stained for 30 min at 4°C with either anti-Mouse IDO1 eFluor® 660 (#50-9473-82, eBioscience) or its isotype control rat IgG2b K eFluor® 660 (#50-4031-82, eBioscience) antibodies (mAbs). After washings, cells were suspended in MACS buffer for further flow cytometry analysis on the LSRFortessa™ (BD Biosciences). Data were analyzed using the FlowJo software (version 9.0.2, Tree Star) to determine the frequency of IDO1 positive cells.

### Ehrlich test

TC-1 cells (1 × 10^5^) were seeded in 24-well plates and incubated in RPMI medium with 10% SFB for 24 h, until reaching 50% of confluence. After this period, fresh medium (1 mL) containing 1 mM of immunomodulators (D-1MT, L-1MT, DL-1MT, or melatonin) was added and the plates were incubated for 36 h. Culture supernatants were assayed for N-formyl-kynurenine as a measure of IDO activity. The supernatants (100 μL) were treated with 50 μL of 30% trichloroacetic acid (TCA), centrifuged for 10 min at 3,000 g, and the supernatant was incubated at 52°C for 30 min to hydrolyze N-formyl-kynurenine to kynurenine. A triplicate of each sample (80 μL) was aliquoted into a 96-well plate. Standards were prepared as serially diluted kynurenine from 1,000 μM in TCA-treated media. Freshly prepared Ehrlich's reagent (80 μL) (2 g of 4-Dimethylamino-benzaldehyde in 100 mL of glacial acetic acid) was added to each well and plate was incubated for 15 min at room temperature in the dark. The plates were read in a spectrophotometer at 492 nm.

### Wound healing assay

TC-1 cells (5 × 10^5^) were seeded in 24-well plates and cultured for 24 h until reaching 95% of confluence. The monolayers were then carefully scratched with the aid of a 200 μL pipette tip followed by the addition of fresh culture medium containing 1 mM of immunomodulators. Cells were photographed after appropriate incubation times using a light microscope.

### Cell adhesion assay

TC-1 cells were cultured until reach 90% of confluence, and then were harvested with trypsin and washed with RPMI 1640 medium supplemented with 10% FBS and 50 U/mL penicillin/streptomycin. Cells were resuspended in fresh culture media containing 1 mM of immunomodulators, and then were seeded in 24-well plates at a concentration of 1 × 10^5^ cell/well. Cells were incubated at 37°C and 5% CO^2^ for 2 h. Next, the plate was placed on ice and cells were washed twice with ice-cold PBS to removing the non-adherent cells. Cells were then fixed in ice-cold methanol for 10 min and stained for 20 min with crystal violet solution (0.5% w/v, made in 25% methanol). Finally, plates were carefully rinse in distillated water until color no longer coming off in rinse. Cells were photographed in EVOS® FL Cell Imaging System (Thermo Fisher Scientific). The percentages of cells adhesion were measured by numbers of cells per field.

### Cell viability

TC-1 cells (1 × 10^5^) were seeded in 24-well plates and incubated in RPMI medium with 10% SFB for 24 h, until reaching 50% of confluence. After this period, fresh medium, containing 1 mM of immunomodulators was added, and the plates were incubated for additional 24 h. Next, cell cytotoxicity was assessed using ethidium bromide (EB) incorporation in combination with acridine orange (AO) staining as described previously (32). Images were acquired in EVOS® FL Cell Imaging System (Thermo Fisher Scientific) and the numbers of death cells were counted per field.

### Mice and TC-1 tumor cell challenge

Wild type (WT) C57BL/6 mice, aged 8–10 weeks, were purchased from the Department of Parasitology of Institute of Biomedical Sciences and the Faculty of Veterinary Medicine of the University of São Paulo. The IDO1 gene (IDO^−/−^) knocked mice were supplied by the animal facility unit of the Department of Immunology of the University of São Paulo. All procedures for manipulation, immunization and euthanasia were approved by the ethics committee for animal experimentation (protocol number CEUA 050/2014) and followed the standard rules approved by the National Council for Control of Animal Experimentation (CONCEA). The tumor cells transplantation was performed as previously described (29), at a concentration of 1 × 10^5^ cells/100 μL/animal on 0 day. Mice were considered as tumor-bearing when tumors became palpable (7–10 day) and were sacrificed when tumors exceeded 15 mm in “L” diameter.

### Immunization and immunomodulators

The therapeutic gDE7-based vaccine was administered following a regimen of two subcutaneous doses with a week interval, as previously described (28). Each dose contained 30 μg of the gDE7 protein, diluted in saline solution (total volume of 100–200 uL) and inoculated in the right rear flank region of mice on 7 and 14 day. In addition to the vaccine, mice were also treated with 1MT and/or melatonin for 4 weeks every 48 h, starting on 9 day. The D-1MT isomer and the racemic mixture DL-1MT were given in concentrations of 10 mg/animal dissolved in a mixture of 0.5% tween-80, 0.5% methyl cellulose (33) and sterile milli Q water, being administered 100 μL/animal per gavage. Melatonin was given at a concentration of 0.2 mg/200 μL/animal, dissolved first in DMSO (1%) and subsequently in apyrogenic saline solution, being administered intraperitoneally.

### Blood, spleen, and tumor microenvironment analyses

Blood, spleen and tumor (when applicable) from naïve and tumor-bearing WT or IDO1^−/−^ mice were used to analyze the frequency of different cell types. For blood samples, peripheral blood mononuclear cells were collected in heparin-containing vials. Cells were treated with ACK Lising Buffer (BioSource International) for lysis of red blood cells. Next, cells were washed with RPMI medium with 10% FBS (R10) and distributed in 96-well U-bottom plates for further staining. For spleen and tumor samples, mice were euthanized, and spleens and tumors were removed aseptically. Spleens were macerated with the aid of a syringe plunger, suspended in R10, filtered in a 70 μm cell strainer (Easy strainer Greiner Bio One) and treated with ACK lysis buffer. Subsequently, cells were washed and distributed in 96-well U-bottom plates for further staining. Tumor masses were minced with scissors and submitted to enzymatic digestion with 0.22 u/mL of collagenase D (#11088866001, Roche Diagnostics) at 37°C for 1 h, stirring gently every 10 min. After the incubation period, the enzyme was inactivated with 5 mM EDTA at room temperature for 5 min. Then, the samples gentle resuspended in R10 and filtered on a 70 μm cell strainer (Easy strainer Greiner Bio One). After centrifugation, the pelleted cells were resuspended in R10 and filtered on a 40 μm cell strainer. Then, cells were centrifuged, the pellet were resuspended in R10 and distributed in 96-well U-bottom plates for further staining. The following mAbs were used to discriminate different types of cells: anti-CD45-PerCP-Cyanine5.5 (#103131, Biolegend), anti-CD4-FITC (#553651, BD Pharmingen), anti-CD25-APC (#17-0251, eBioscience), anti-Foxp3-PE (#12-4771-80, eBioscience), anti-CD11b-Alexa Fluor 700 (#101222, BioLegend), anti-Gr-1-PE (#553128, BD Pharmingen), anti-Ly6C-Alexa Fluor 488 (#53-5932-82, eBioscience), anti-Ly6G-PE (#551461, BD Pharmingen), anti-CD11c-PE (#553802, BD Pharmingen), anti-MHC-II-FITC (#553605, BD Pharmingen), anti-F4/80-BV605 (#123133, BioLegend), anti-IDO1 eFluor® 660 (#50-9473-82, eBioscience), isotype control rat IgG2b K eFluor® 660 (#50-4031-82, eBioscience). For FoxP3 intracellular staining, we used the Foxp3 Transcription Factor Staining Buffer Set (#00-5523-00, eBioscience). For intracellular IDO-1 staining, we used the Intracellular Fixation and Permeabilization Buffer Set (#555028, BD Cytofix/CytopermTM Plus). Cells were characterized according to the following parameters: dendritic cells (CD45+, CD11chigh, MCH-IIhigh), macrophages (CD45+, MCH-II+, CD11b+, F4/80+), inflammatory monocytes (CD45+, CD11bint, Ly6Chigh, Ly6G- or CD45+, CD11bint, Gr1int), resident monocytes (CD45+, CD11bint, Ly6Cint, Ly6G-), MDSC (CD45+, CD11bhigh, Ly6Cint, Ly6G+ or CD45+, CD11bhigh, Gr1high), Treg (CD45+, CD4+, CD25+, FoxP3+). The gate strategy could be observed in the Supplementary Figure S1. Cells were acquired by LSR Fortessa™ (BD Biosciences) flow cytometer and data were analyzed using the FlowJo software.

### Intracellular cytokine staining

Intracellular IFN-γ staining was performed as previously described (29). The mouse peripheral blood mononuclear cells were collected in heparin-containing vials14 days after the last gDE7 immunization (28 day).

### Statistical analysis

Statistical analyses were performed using Prism (GraphPad) software. The analysis was performed using the unpaired *T*-test, One-Way ANOVA or Two-Way ANOVA and the results confirmed through multiple comparisons by Tukey's test. Values of *p* < 0.05 were considered significant.

## Results

### TC-1 cells express IDO

**Figure 1:**
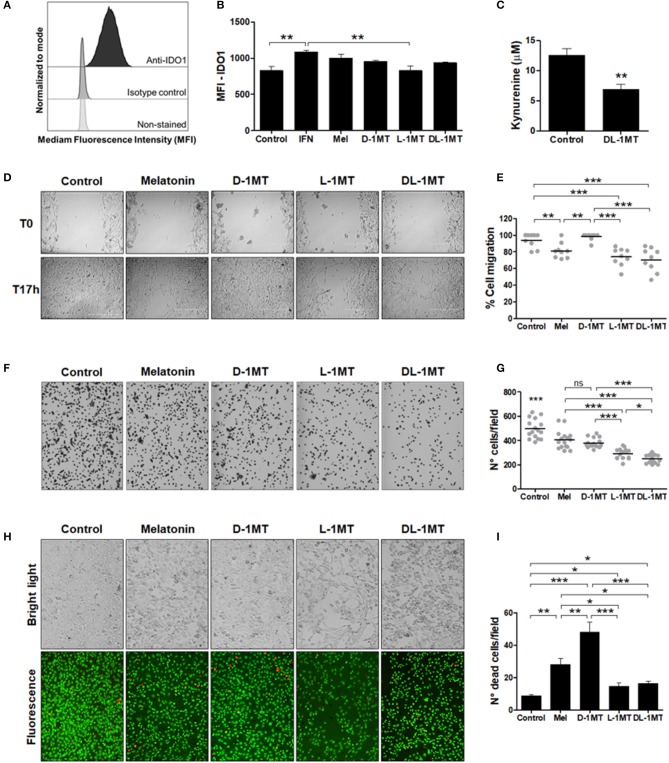
IDO1 expression and the effects of melatonin and IDO inhibitors on TC-1 cells migration and adhesion. **(A)** IDO1 expression measured with anti-IDO1 antibody staining and flow cytometry analysis. Isotype control and non-stained cells were used as negative controls for IDO1 expression and cellular auto-fluorescence, respectively. **(B)** Median fluorescence intensity (MFI) of IDO1 expression in TC-1 cells measured by flow cytometry. Cells were treated with IFN-γ (50 u/mL), or melatonin (1 mM), or 1MT compounds (D-1MT, L-1MT, DL-1MT) (1 mM) for 24 h. Cells in culture media without immunomodulators (vehicle) are shown as reference controls. Data representative of two independent experiments performed in triplicates. **(C)** Ehrlich test performed to measure kynurenine concentrations in TC-1 cell supernatants after treatment with DL-1MT (1 mM) for 24 h. Data representative of two independent experiments performed in triplicates. Significance was determined by unpaired Student's *t*-test. **(D-E)** The effects of 1MT and melatonin (Mel) on TC-1 cells migration. **(D)** The bright-field microscopy imaging of TC-1 cells submitted to different stimulus at the beginning of the test (T_0_) and 17 h later (T_17h_). Cells kept in culture medium were used as a reference control. **(E)** Panels on the right indicate the quantification of the percentage of migratory cells through the cell layer wound healing assay after measurement of uncovered areas at the T_17h_ in relation to T_0_. Data of three independent experiments performed in triplicates. **(F)** Representative bright-field microscopy images of adherent TC-1 cells 2 h after addition of the tested immunomodulators. **(G)** Adhesion of TC-1 cells in the presence of immunomodulators (1 mM) 2 h after seeding 24-well plates. Data representative of two independent experiments performed in triplicates. **(H)** Representative bright-field microscopy images (upper images) and fluorescence microscope images (botton images) to demonstrate cell density (cell proliferation) and cell viability, respectively, after cell growth in the presence of the different immunomodulators. **(I)** The graph represents the number of dead cells per field. Cells treated with culture media were included as controls. Data representative of two independent experiments performed in triplicates. All data are presented as means ± SEM. Statistical significance: ^*^p < 0.05, ^**^p < 0.01, and ^***^p < 0.001 by ANOVA. (ns) Non-significant. When not signaled, ^*^ represents the statistical significance of one experimental group in relation to all others.

Usually IDO expression in murine tumor cells is observed after transfection of cells with IDO1 encoding viruses or after genetic manipulations (33). Here, we verified that, in contrast to other cell lines, the TC-1 cell line express IDO constitutively. IDO expression in TC-1 cells was demonstrated by flow cytometry using an isotype control antibody as a comparative control (Figure 1A). IDO in TC-1 cells was upregulated by IFN-γ but not by melatonin, D-1MT, L-1MT, and DL-1MT (Figure 1B). In addition, TC-1 cells accumulate kynurenine in culture supernatants, which decreased significantly in the presence of DL-1MT (Figure 1C). These results indicate that IDO is enzymatically active in TC-1 cells.

### Melatonin and 1MT have direct effects on TC-1 cells migration, adhesion and viability

We next evaluated whether melatonin and IDO inhibitors would have a direct effect on the *in vitro* growth of the TC-1 tumor cells. With this purpose, we carried out wound healing assays for assessment of cell migration. As shown in Figures 1D,E, melatonin reduced the migratory behavior of TC-1 cells and similar effects were observed in cells treated with L-1MT and DL-1MT. Interestingly, D-1MT did not show any significant effect on migration of TC-1 cells. We also measured the attachment of the TC-1 cells to a plastic surface and all immunomodulators caused a partial impairment of the cell adhesion behavior when compared with untreated cells (Figures 1F,G). No difference was observed between cells treated with melatonin and D-1MT, which decreased cell adhesion by approximately 20%. The racemic mixture of 1MT isomers reduced approximately 50% of cell adhesion, whereas L-1MT decreased cell adhesion by approximately 36% (Figure 1G). Additionally, melatonin, L-1MT and DL-1MT decreased cell proliferation capacity while melatonin and D-1MT were more cytotoxic than L-1MT and DL-1MT (Figures 1H,I). Taken together, these results demonstrate direct effects of melatonin and 1MT derivates on TC-1 cell behavior.

### IDO expression in immune cells increases in the course of tumor growth and impairs the antitumor effects of gDE7-based immunotherapy

**Figure 2:**
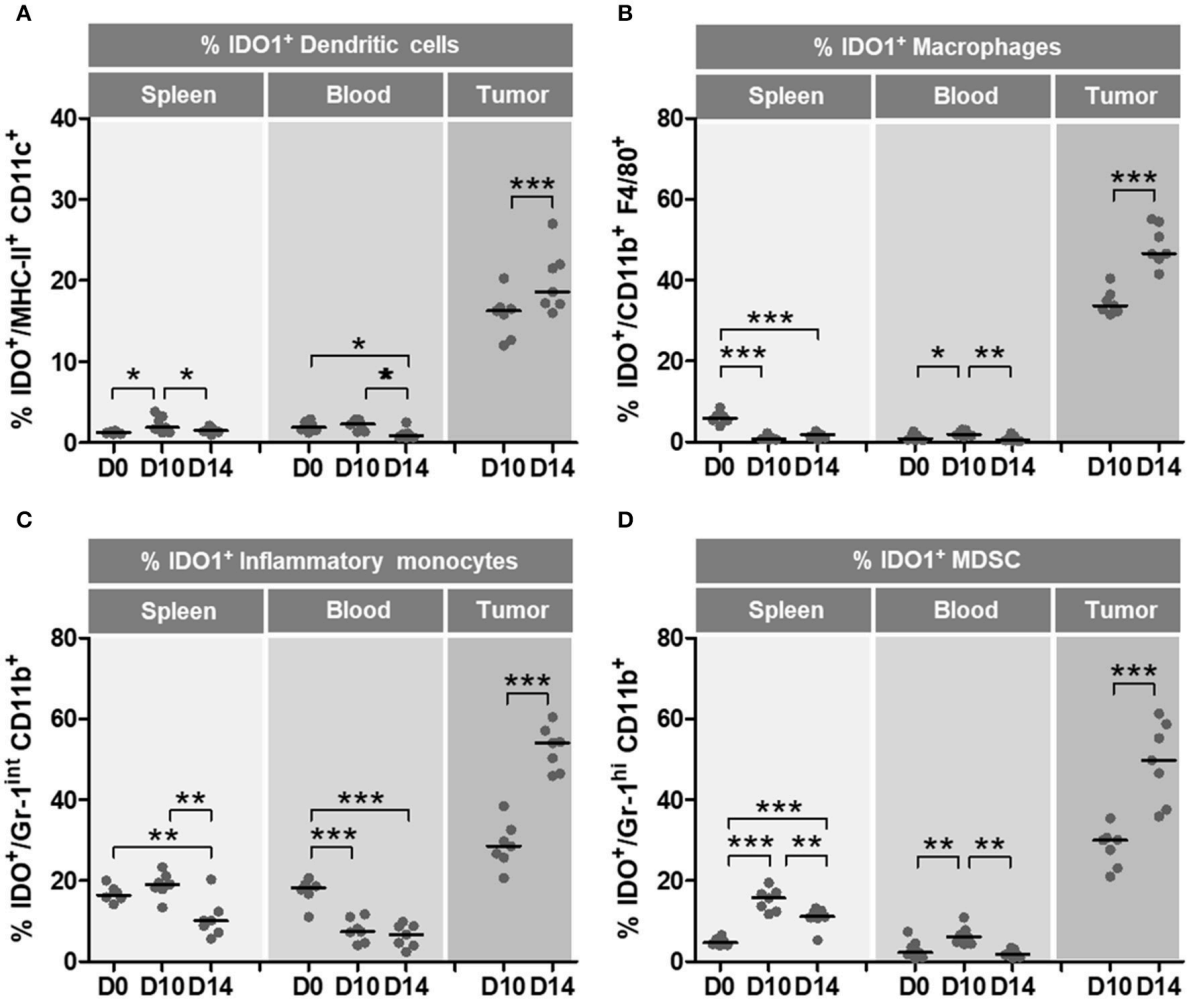
Immune microenvironment cells increase IDO1 expression during oncogenesis. Wild-type (WT) C57BL/6 mice (*n* = 7) were subcutaneously injected with 1 × 10^5^ TC-1 cells and tumors were evaluated at 10 and 14 day after the tumor cell transplantation. Naïve mice were used as a reference of physiological parameters. The frequencies of DCs, macrophages, inflammatory monocytes and MDSC were evaluated in spleens, blood and tumor tissues. IDO1 expression was measured by intracellular staining and flow cytometry analyses. **(A–D)** Frequency of IDO1-expressing immune cells at 0 day (D0–naïve mice), 10 day (D10) and 14 day (D14) after TC-1 transplantation. Parameter used to characterize the different cell populations: **(A)** DCs (CD45^+^, CD11c^high^, MCH-II^high^), **(B)** macrophages (CD45^+^, MCH-II^+^, CD11b^+^, F4/80^+^), **(C)** inflammatory monocytes (CD45^+^, CD11b^int^, Ly6C^high^, Ly6G^−^), **(D)** MDSC (CD45^+^, CD11b^high^, Ly6C^int^, Ly6G^+^). Data representative of two independently performed experiments. Statistical significance: ^*^p < 0.05, ^**^p < 0.01, and ^***^p < 0.001 by ANOVA.

To evaluate the role of IDO1 in the *in vivo* growth of TC-1 cells, expression of IDO1 was measured in DCs, macrophages, inflammatory monocytes and MDSCs from spleen, blood and tumor masses at 10 and 14 days after tumor cell transplantation in wild type mice. As shown in Figure 2, there was a substantial increase of IDO1 expression in immune cells at the tumor microenvironment over time. Comparing 0 and 10 day with day 14 post TC-1 transplantation, we observed a concomitant decrease in the number of IDO1-expressing DCs (Figure 2A), macrophages (Figure 2B), inflammatory monocytes (Figure 2C) and MDSCs (Figure 2D) in spleen and blood of TC1-grafted mice, which suggests that these cells are migrating to the tumor site. These observations emphasize that IDO1 expression by immune cells in the tumor microenvironment contributes to the immunosuppressive environment that may affect the efficacy of immunotherapies.

**Figure 3:**
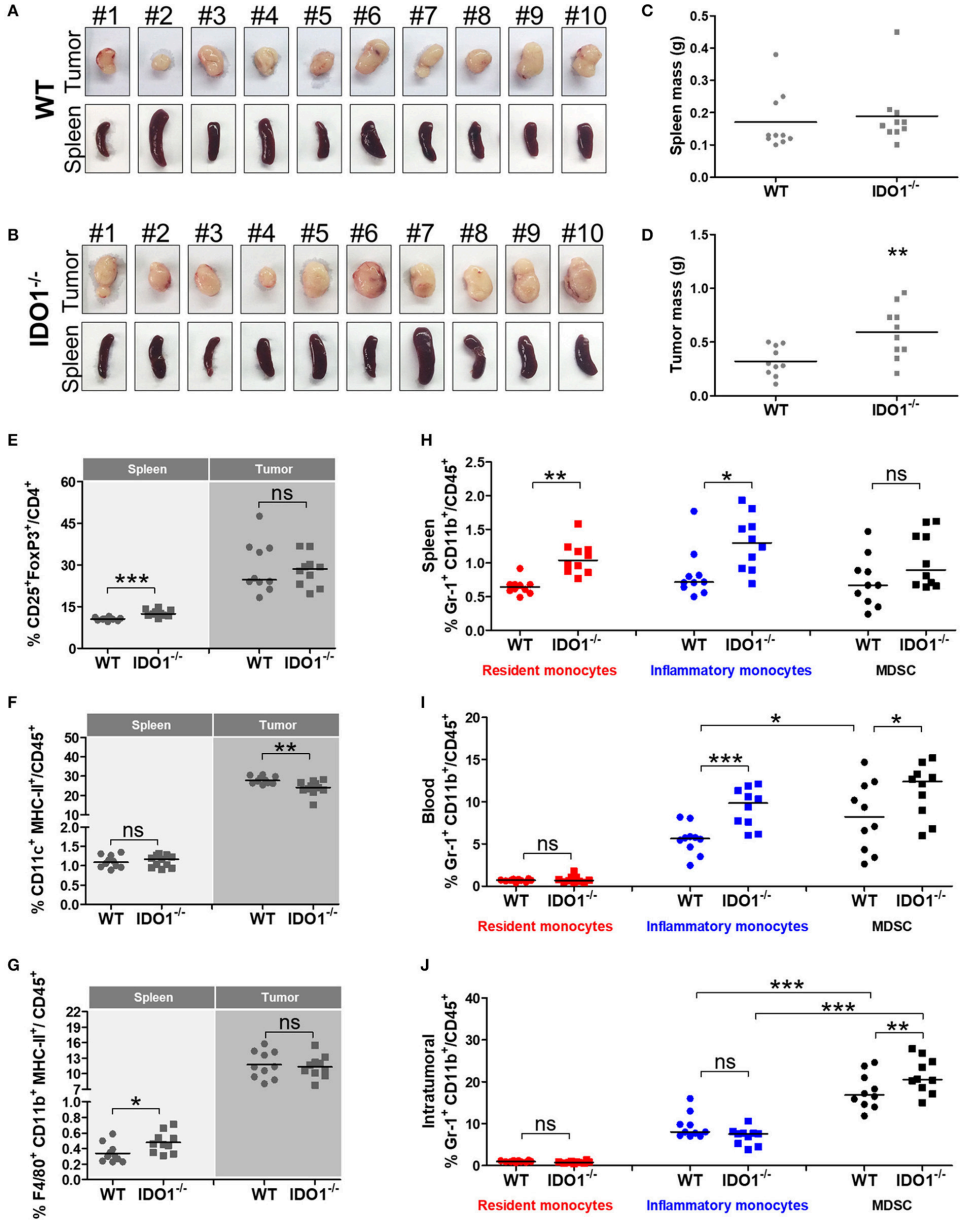
The absence of IDO1 expression modifies the frequency of immunological cells in distinct mice tissues. Images of tumor masses and spleen from wild-type (WT) **(A)** and IDO^−/−^**(B)** mice subcutaneously injected with 1 × 10^5^ TC-1 cells and euthanized 21 days later (*n* = 10 per group). Comparative analyses of **(C)** spleen and **(D)** tumor masses of WT and IDO^−/−^ mice. Comparative analyses of **(E)** Treg, **(F)** DCs and **(G)** macrophages in spleens and tumor tissues of WT and IDO^−/−^ mice. Comparative analyses of resident monocytes, inflammatory monocytes and MDSC in **(H)** spleens, **(I)** blood and **(J)** tumor tissues of WT and IDO^−/−^ mice. Statistical significance: ^*^p < 0.05, ^**^p < 0.01, and ^***^p < 0.001 by ANOVA or unpaired Student's *t*-test. (ns) Non-significant.

To further evaluated the role of IDO1 in the growth of tumor cells and tissue-specific microenvironment, we grafted the TC-1 cells in IDO1^−/−^ mice and measured the presence of macrophage, DCs and immunosuppressive cells in spleens and tumor tissues 21 post tumor cell transplantation (Figures 3). Interestingly, larger tumors were observed in IDO1^−/−^ mice regarding the parental mouse strain, although no difference was observed in the spleens (Figures 3A–D). Moreover, although Treg cell population was increased in the spleen of the IDO1^−/−^ mice, the frequency of these cells in the tumor microenvironment was the same in both mouse strains (Figure 3E). In contrast, the capability of DCs to migrate to the tumor site was reduced in IDO^−/−^ mice (Figure 3F), while there was no difference in the frequency of macrophages frequency in the spleen and tumor from both mouse strains (Figure 3G). Interestingly, while IDO1^−/−^ mice had a higher frequency of MDSCs in the blood and tumors than the C57Bl/6 mice, we observed a higher frequency of resident monocytes in the spleens and a higher frequency of inflammatory monocytes in both spleens and blood of IDO^−/−^ mice (Figures 3H–J). However, at 21 day post TC-1 cell engraftment, no differences in the frequency of these cells were observed in the tumor microenvironment (Figure 3J). It is important to highlight that cells from IDO^−/−^ mice did not express IDO1 (data not shown) and the only source of this enzyme was the transplanted TC-1 cells.

**Figure 4:**
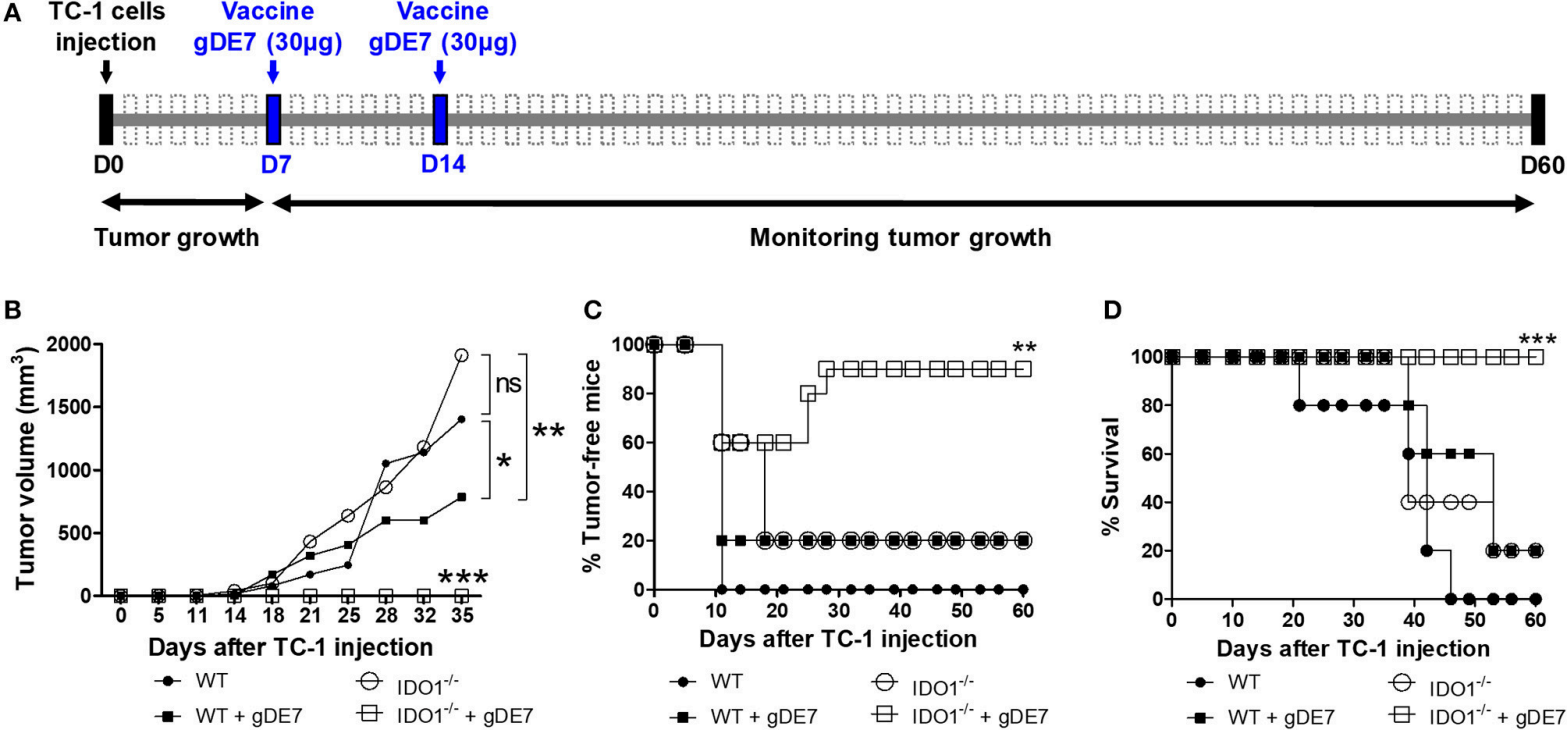
IDO expression impairs the antitumor effects of gDE7-based immunotherapy. **(A)** Wild-type (WT) and IDO^−/−^mice were subcutaneously injected with 1 × 10^5^ TC-1 cells and immunized with two doses (7-day interval) of gDE7 (30 μg per animal) via subcutaneous (s.c.) route. Tumor growth was monitored until 60 day, when the animals were euthanized. Antitumor effects were measured by **(B)** tumor volume, **(C)** the percentage of tumor-free mice and **(D)** the percentage of mice survival. Vaccinated IDO^−/−^ mice were significantly different than all other groups, highlighting that the gDE7-dependent antitumor effects are enhanced by inactivation of host IDO gene expression. Data from two identical experiments (*n* = 5) were pooled and analyzed by ANOVA or by Kaplan-Meyer test (exclusively for survival assay). ^*^p < 0.05, ^**^p < 0.01, and ^***^p < 0.001. (ns) Non-significant. When not signaled, ^*^ represents the statistical significance of one experimental group in relation to all others.

Subsequently, we evaluated the impact of IDO1 expression on the efficacy of gDE7-based immunotherapy in C57Bl/6 and isogenic IDO1^−/−^ mice. Seven days after TC-1 injection, mice were immunized at a suboptimal conditions (28, 29). The vaccine was subcutaneously administered twice (7-day interval), starting 7 days after TC-1 cells challenge, a time point in which the tumors became palpable (Figure 4A). Although we observed a tendency of tumors to be higher in IDO1-deficient animals, there was no statistical difference concerning the tumor growth kinetics up to 35 days, in both WT and IDO^−/−^ not immunized mice, when tumors achieve a diameter of approximately 13-14 mm (Figure 4B). In contrast, the protective immunity conferred by gDE7 was significantly enhanced in IDO^−/−^ mice regarding WT mice (100% and 20% survival, respectively) (Figures 4B–D). These results demonstrated that although the absence of IDO1 did not impair TC-1 cells *in vivo* growth, IDO1 have a dramatic effect in the modulation of protective antitumor immune responses elicited by animals submitted to the immunotherapy.

### Combination of melatonin and 1-DL-MT improves the efficacy of gDE7-based immunotherapy

**Figure 5:**
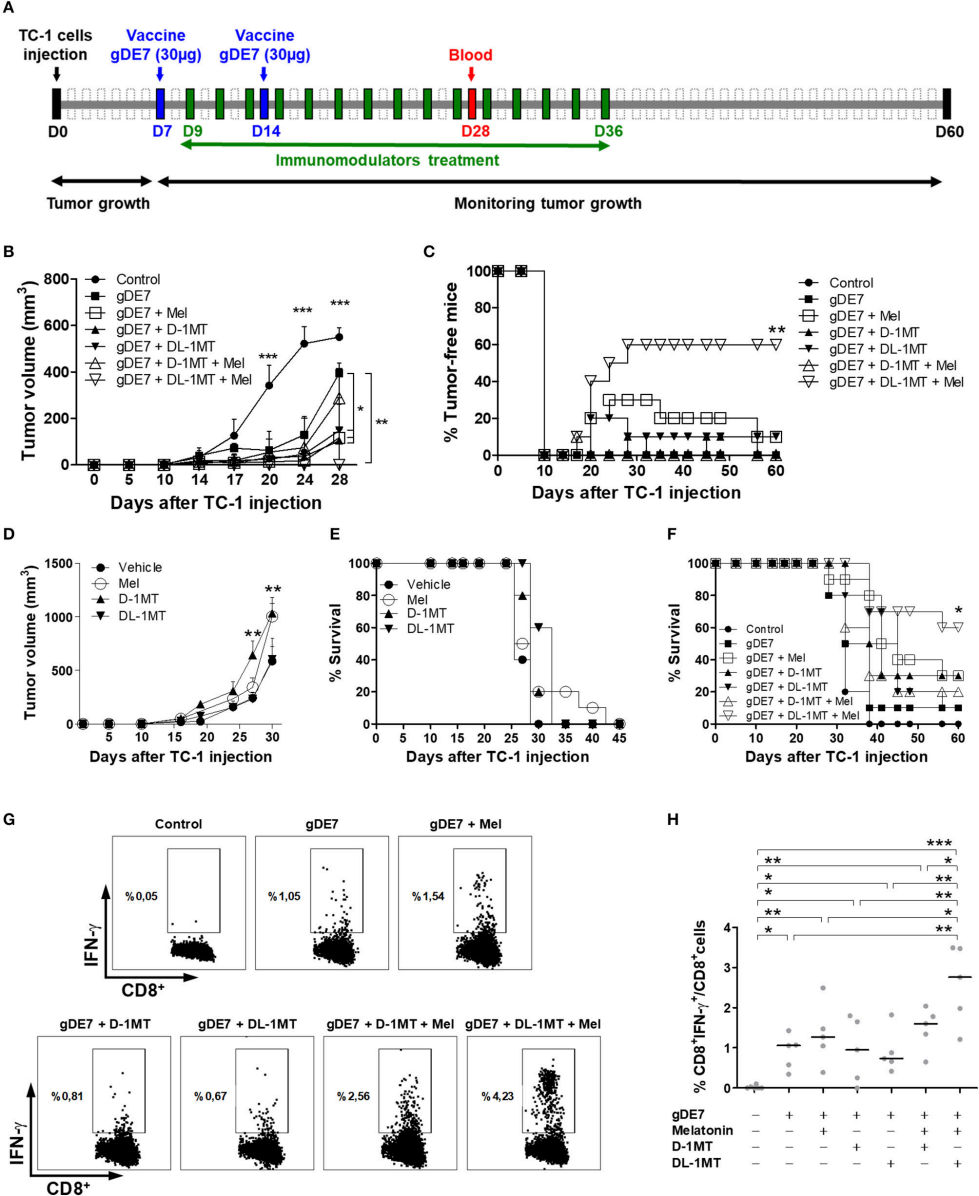
Co-administration of melatonin and DL-1MT synergistically enhances the antitumor effects of gDE7-based vaccine in immunized mice. **(A)** WT mice were subcutaneously injected with 1 × 10^5^ TC-1 cells and immunized with two doses (7-day interval) of gDE7 (30 μg per animal) via the s.c. route (day 7-D7 and day 14-D14). The treatment with melatonin (0.2 mg per animal, intraperitoneally) or 1MT compounds (10 mg per animal through gavage) started 2 days after the first vaccine dose (day 9-D9) and every 48 h till 36 day (D36). Untreated and unvaccinated mice were considered control groups. The tumor growth was monitored for 60 days (D60). **(B,C,F)** An enhanced antitumor effect induced by gDE7 was observed after its combination with a single immunometabolic adjuvant. However, the association of melatonin (Mel), DL-1MT and gDE7 resulted in maximal antitumor effects regarding **(B)** tumor size, **(C)** tumor eradication (tumor-free-mice) and **(F)** mice survival. To analyze the effect of immunometabolic adjuvants on tumor growth, C57BL/6 mice were subcutaneously injected with 1 × 10^5^ TC-1 cells and treated with 10 mg/animal of D-1MT or DL-1MT or 0.2 mg/200 μL/animal of melatonin for 4 weeks every 48 h, starting on 9 day. One group received apyrogenic saline and the 1MT vehicle as a control. Antitumor effects for each tested group was evaluated by **(D)** tumor volume and **(E)** the percentage of survival. The tumor growth was monitored until 45 day, when the animals were euthanized due to tumor size. **(G,H)** Blood samples were harvest at 28 day (D28) post TC-1 cells injection and analyzed for the frequencies of activated CD8^+^ T-cells (CD8^+^IFN-γ^+^ T-cells) by flow cytometry. **(G)** Plots of circulating E7-specific IFN-γ producing CD8^+^ T cells. **(H)** Percentages of circulating E7-specific CD8^+^IFN-γ^+^/CD8^+^ T cells in each tested mice groups. Data from two identical experiments (*n* = 5) were pooled and analyzed by ANOVA or by Kaplan-Meyer test (exclusively for survival assay). ^*^p < 0.05, ^**^p < 0.01, and ^***^p < 0.001. (ns) Non-significant. When not signaled, ^*^ represents the statistical significance of one experimental group in relation to all others. Data represent means ± SD from one representative of two independently performed experiments (*n* = 5) with comparable results.

We next evaluated if association of a suboptimal vaccine regimen combined with melatonin and1-MT would enhance the antitumor immunity elicited in mice challenged with TC-1 cells. The treatment with immunometabolic adjuvants started 2 days after the first gDE7 dose (9 day) and finished on 36 day (Figure 5A). As previously shown, mice treated only with the vaccine (gDE7 group) showed partial tumor control (Figure 5B) and no tumor-free record (Figure 5C). Similarly, mice treated only with the immunomodulators did not exhibit significant tumor growth control (Figures 5D,E). Meanwhile, the combination of the immunotherapy and the immunometabolic adjuvants promoted a significant increase in the antitumor protective immunity (Figures 5B,C,F). Mice treated with gDE7 and melatonin, D-1MT or DL-1MT showed significant tumor growth delay compared with the gDE7-treated group (Figure 5B). Lower protective values were observed when the numbers of tumor-free mice were considered, with 10% tumor-free mice for animals treated with gDE7 and melatonin, or gDE7 and DL-1MT, and none for those animals treated with gDE7 or gDE7 plus D-1MT (Figure 5C). However, the combination of melatonin and DL-1MT, but not with D-1MT, synergistically enhanced the anti-tumor protective effects conferred by gDE7, leading to a complete antitumor protection in 60% of challenged mice (Figures 5C,F) at the end of the observation period. Collectively, these results demonstrated that melatonin improves the performance of a cancer immunotherapy when combined with DL-1MT.

Finally, we next evaluated the presence of circulating cytotoxic CD8^+^ T cells, which have a pivotal role on the elimination of the tumor cells. The results indicated an increased number of IFN-γ producing CD8^+^ T cells in gDE7-immunized mice treated or not with melatonin or 1MT compounds compared to non-immunized group (Figures 5G,H). Noticeably, E7-specific CD8^+^ T cells isolated from mice treated with both melatonin and DL-1MT produced higher levels of IFN-γ in response to stimulation with the E7-derived peptide compared to the other immunization groups (Figure 5H). Taken together, the present data demonstrated that the association of melatonin and DL-1MT synergistically enhance the induction of protective immune responses and increase the antitumor immunity elicited in immunized mice.

## Discussion

Considering the increase association of IDO1 expression and HPV-induced malignancies, incorporation of two immunometabolic adjuvants, melatonin and IDO1 inhibitors, to an anti-cancer vaccine resulted in enhanced *in vivo* antitumor effectiveness without visually noticeable side effects usually observed with other anti-cancer treatments. The combination of IDO inhibitors to the immunotherapy clearly increased the anti-cancer effects by reducing the negative impact of IDO1 expression on the protective immunity induced by the vaccine. The incorporation of melatonin to the proposed immunization regimen was supported by previous evidences that, in addition to its oncostatic properties (34), it negatively regulates IDO1 expression and its synthesis can be driven by 1MT, a classical IDO1 inhibitor (16). The results demonstrate that the unprecedented combination of melatonin and IDO1 inhibitors (particularly DL-1MT) improves the performance of the anti-cancer vaccine, leading to enhanced antitumor protection and activation of E7-specific CD8^+^ T cell response. Altogether, the present evidences further support the beneficial effects of immunometabolic adjuvants to the treatment of tumors, particularly those associated with papillomaviruses, and support further investigations under clinical conditions.

A hierarchical profile between IDO1 expression have been observed in different cancer types, whereupon endometrial and cervical cancer had the highest and most frequent IDO1 expression (35, 36). In cervical cancer, IDO^+^ cells were often located at the periphery of tumor nodules, surrounded by IFN-γ producing T lymphocytes (35, 36). The involvement of IDO1 in the mounting of an immunosuppressive microenvironment in HPV-associated cervical cancer was first reported in 2008 by Kobayashi and collaborators, who showed that the numbers of IDO1-expressing immune cells significantly increased from normal cervix condition to the cancerous state (37). Notably, by measurement of tryptophan and kynurenines metabolites in serum samples of cervical cancer patients, enzymatically active IDO1 was associated with a poor clinical outcome (38). Indeed, several pathological parameters, such as tumor size, lymph node metastasis and advanced disease stage, emphasize the role of IDO1 expression in promotion of tumor growth and highlight the potential positive impacts of IDO1 inhibition in the fate of tumor treatments (38). Regarding the contribution of IDO1 to an immunosuppressive milieu, microenvironment analyses revealed higher IDO1 expression in dermal DCs from grafted skin cells expressing HPV-16 E7 oncoprotein than nontransgenic control skin cells. In addition, treatment of mice engrafted with HPV oncoprotein-expressing cells with DL-1MT promoted skin graft rejection (39).

The increased frequencies of IDO1-expressing cells, such as DCs, macrophages, inflammatory monocytes and MDSC, in mice transplanted with TC-1 cells indicated that the use of IDO1 inhibitors would improve tumor growth control. We initially used IDO1-deficient mice (IDO1^−/−^) to evaluate the influence of IDO1 expression on tumor growth. Interestingly, lack of IDO1 expression in the host did not impair the *in vivo* growth of TC-1 cells. This outcome has also been observed in the melanoma (B16F10 cells) tumor model (33) and in the azoxymethane-induced colon tumor model (40). Nonetheless, lack of IDO1 expression positively impacted the protective antitumor immunity elicited in mice immunized with gDE7. This phenomenon could be partially explained by the greater inflammatory potential of these animals when compared to WT mice, since IDO1^−/−^ tumor-bearing mice showed higher frequencies of resident monocytes in spleens and inflammatory monocytes in both spleens and blood. Indeed, in a pulmonary model of paracoccidioidomycosis, the absence of IDO1 expression led to a higher influx of activated inflammatory cells into the lungs, which promoted an increased expansion of T cells (41). Moreover, IDO1 knockout mice showed increased pro-inflammatory cytokines expression and decreased Treg cells in a colon tumor mouse model (40). Thus, the present results add a new piece of evidence that IDO1-targeting therapy could improve antitumor therapies by reprogramming inflammatory cells.

Since cancer is a multifactorial disease that arises from alterations in different physiological processes, multidrug anti-cancer treatments may reach a better outcome at clinical conditions. Currently, passive immunotherapies, based on mAbs targeting different cellular checkpoint controllers, had changed the landscape of cancer treatment, such as those blocking cytotoxic T lymphocyte-associated antigen 4 (CTLA-4) and programmed cell death 1 (PD-1) or its ligand PD-L1 (42). However, treatment of solid tumor still poses a challenge due to the frequent emergence of either innate or acquired resistance (43). Indeed, IDO1-expressing cells promote PD-L1 expression in DCs, which in turn activate Treg cells, while treatment with anti-PD-L1 or anti-PD-L2 or anti-PD-1 can reverse this effect (44). In addition, IDO1 expression has been associated with the neovascularization of tumor metastasis (45). In this scenario, the combination of IDO1 inhibitors with immunotherapies and other anti-cancer drugs seems to be particularly encouraging and emphasizes the relevant role of tumor biology knowledge in the development of more efficient therapies.

Regarding the translational use of IDO inhibitors, different clinical trials are ongoing. Recently, a failure on a phase 3 trial in metastatic melanoma, based on the combination of epacadostat (IDO1 inhibitor) with pembrolizumab (anti-PD-1 antibody), generated a disappointment in the so-called “second generation” of immuno-oncology drugs (clinical trial information: NCT02752074) (25). However, in another trial based on epacadostat plus a multipeptide melanoma vaccine, besides normalized serum kynurenine/tryptophan ratios in most patients, data indicated an enhancement of CD8^+^ T cell infiltration in tumor milieu in patients with melanoma submitted to the combination therapy (clinical trial information: NCT01961115) (26). Interestingly, a phase 2 trial with another class of IDO inhibitor, the indoximod (D-1MT), plus gemcitabine and nab-paclitaxel showed promising results regarding the use of IDO inhibitors for patients with metastatic pancreas cancer (clinical trial information: NCT02077881) (27). In this trial, data indicate increased intra-tumoral CD8^+^ T cell density in biopsies of responder patients submitted to the combination therapy. Overall, these data highlight the importance of the association of antitumor vaccines, and/or immuno-chemotherapy with IDO inhibitors.

We recently showed that the gDE7-based vaccine induces multifunctional E7-specific CD8^+^ T cells with cytotoxic activity as well as expansion of effector memory T cells and activation of mouse and human specialized DC subset capable to promote antigen cross-presentation (29). In the present study, we observed that treatment with one metabolic adjuvant provided enhanced gDE7-mediated antitumor protection but only the combination of melatonin and one IDO inhibitor conferred complete tumor protection. Regarding IDO1 inhibitors, we observed a superior preclinical antitumor activity relative to DL-1MT in side-by-side comparisons to D-1MT, which is the isoform actually under clinical trials (23). Previous evidences indicated that D-1MT was more effective than DL-1MT as an anti-cancer agent and reversed the T cell suppression effect mediated by IDO1-expressing DCs (33). Similarly, recruitment and activation of tumor-infiltrating MDSCs and regulatory T cells, driven by expression of IDO1, could be successfully reversed by D-1MT in mice (19). On the other hand, the anti-cancer effects of DL-1MT has been attributed to capacity to abrogate the anti-proliferative effects of IDO1-expressing mesenchymal stromal cells (46). Additionally, DL-1MT can down-regulates expression of paxillin-family proteins and promotes activation of AHR-driven responses in mesenchymal stromal cells (47) rising a pro-inflammatory signature that may augment the efficacy of cancer immunotherapies. From the point of view of the direct effects of 1MT on TC-1 cells, it is important to notice that 1MT isomers and, its racemic mixture, showed distinct *in vitro* cellular effects. D-1MT proved to be more cytotoxic than the other compounds whereas DL-1MT notably impacted the adhesion of the TC-1 cells, a phenomenon that could be explained by its presumed impact on the disturbance of cytoskeleton proteins (44). Our data brings additional information about the effects of 1MT, showing that besides the modulation of the inflammatory responses, 1MT isomers have also significant effects on the cell behavior that may impact antitumor responses induced by the therapy.

Regarding the fact that 1MT (D-1MT or DL-1MT) can promote partial activation of CD8^+^ T cells, the addition of melatonin to the combined immunotherapy led to increased frequencies of tumor-reactive cytotoxic T lymphocytes capable to clear tumor cells. Available evidence indicates that melatonin enhances human and mice T cells activation (12), and is involved in the regulation immune functions by modulating T cells polarization (48). Therefore, melatonin has been used to synergize immune-activation with conventional cancer treatment modalities, emerging as an important anti-cancer molecule acting at different stages of tumor progression (49). In fact, melatonin acts as an anti-cancer inhibitory molecule targeting anti-proliferative signaling (16), angiogenesis (50), tumor evading mechanisms (48), tumor metastasis (51) and induction of cell death (52). Additionally, melatonin powerfully enhances cisplatin-induced cytotoxicity and apoptosis in cervical cancer HeLa cells *in vitro*, exhibiting cytotoxic, pro-oxidant, and pro-apoptotic actions in this cells line (52).

It is important to highlight that the therapeutic potential of both melatonin and 1MT were observed only when combined with gDE7-based vaccine. Treatment of tumor-bearing mice with melatonin or 1MT without co-administration of gDE7 did not generate significant antitumor protection. In fact, treatment with melatonin or D-1MT promoted faster tumor growth in mice implanted with TC-1 cells. Therefore, the present results demonstrate that the antitumor effects of melatonin and 1MT are restricted to conditions where the drugs are co-administered to animals in combination with an active immunotherapy, which emphasizes their metabolic adjuvant roles.

In conclusion, the present study demonstrates that the combination of melatonin and IDO1 inhibitors display synergistic effects when combined with a tumor-specific immunotherapy and, therefore, represents a new and promising perspective for the control of HPV-associated tumors, and possibly other cancer types.

## Author contributions

AM: Conception and design, development of methodology, acquisition of data, analysis and interpretation of data, writing, review and/or revision of the manuscript, administrative, technical, or material support, and study supervision. BP: Acquisition of data, analysis and interpretation of data, writing, review and/or revision of the manuscript. RLP: Acquisition of data, writing, review and/or revision of the manuscript. PS: Acquisition of data. RP: Acquisition of data. KR: Acquisition of data. TB: Acquisition of data. LA: Writing, review and/or revision of the manuscript. EdA: Acquisition of data. VC: Administrative, technical, or material support, review and/or revision of the manuscript. LF: Conception and design, interpretation of data, review and/or revision of the manuscript, administrative, technical, or material support, study supervision.

### Conflict of interest statement

The authors declare that the research was conducted in the absence of any commercial or financial relationships that could be construed as a potential conflict of interest.

## Supplementary material

Supplementary Figure S1 Gating strategy for the evaluation of immune cells in blood, spleen and tumor microenvironment. Doublets were initially excluded from analysis by FSC and SSC parameters. Cells were gated by the expression of CD45^+^ and subsequently separated according to cell type specific markers. Dendritic cells and macrophages were distinguished by CD11c^high^ MCH-II^high^ and MCH-II^+^ CD11b^+^ F4/80^+^ expression, respectively. Resident monocytes were characterizedby the expression of CD11b^int^ Ly6C^int^ Gr1^−^, inflammatory monocytes by the expression of CD11b^int^ Ly6C^high^ Ly6G^−^ or CD11b^int^ Gr1^int^ and MDSC by the expression of CD11b^high^ Ly6C^int^ Ly6G^+^ or CD11b^high^ Gr1^high^. Inflammatory myeloid cells were considered tolerogenic when IDO expression was detected intracellularly. For Treg cells analysis cells were separated by the expression of CD4^+^ followed by gating on CD25^+^ FoxP3^+^. Finally, the antitumor specific response were caracterized by E7-specificIFN-γ^+^ producing CD8^+^ T cells.
