# Supplementary figures and images for: The Combined Use of Melatonin and an Indoleamine 2,3-Dioxygenase-1 Inhibitor Enhances Vaccine-Induced Protective Cellular Immunity to HPV16-Associated Tumors
Source: Front Immunol. 2018 Aug 22;9:1914. doi: 10.3389/fimmu.2018.01914 (PMC6113858; doi:10.3389/fimmu.2018.01914)

Figure S1

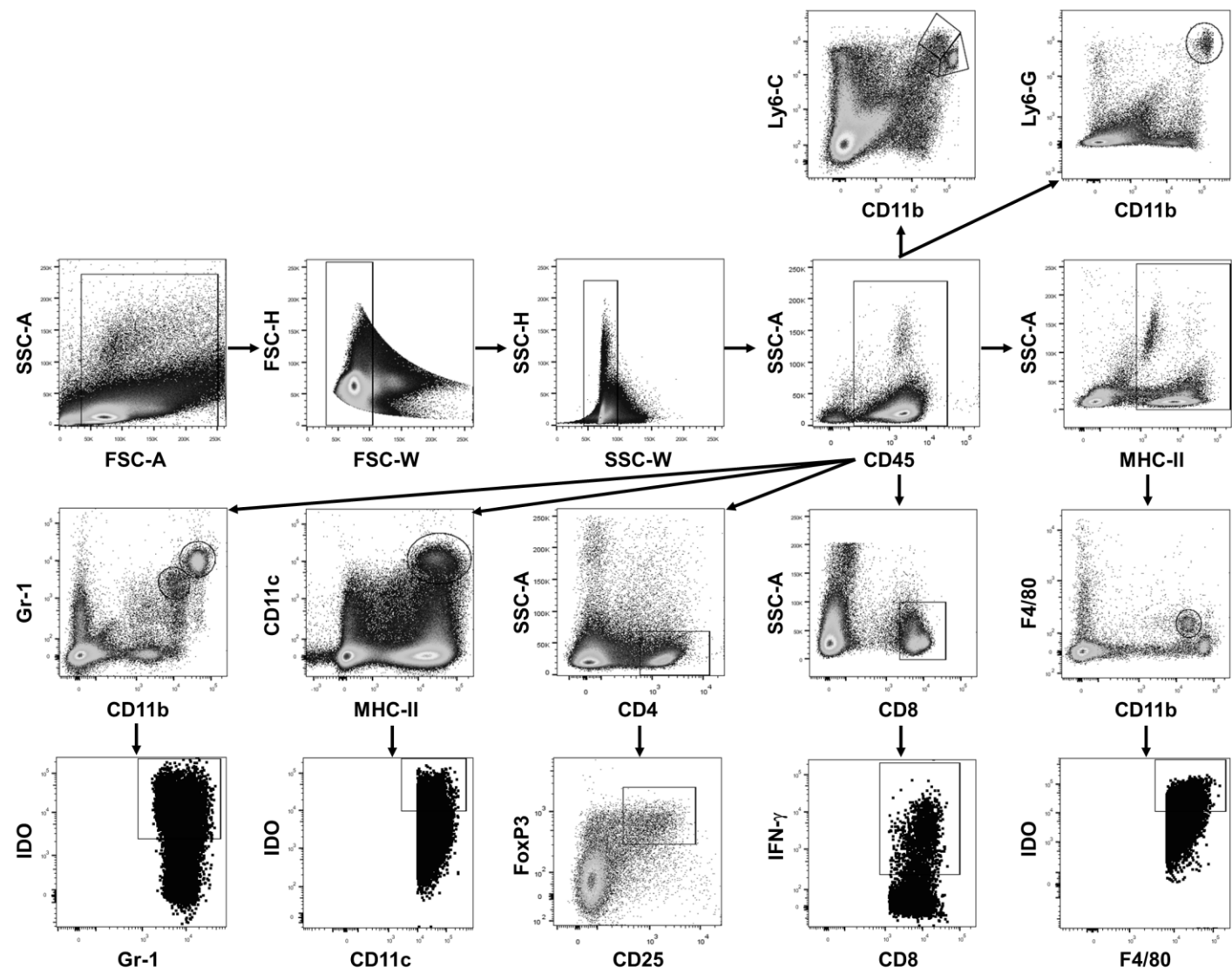

Supplement: Supplementary Figure S1 — Gating strategy for the evaluation of immune cells in blood, spleen and tumor microenvironment. Doublets were initially excluded from analysis by FSC and SSC parameters. Cells were gated by the expression of CD45+ and subsequently separated according to cell type specific markers. Dendritic cells and macrophages were distinguished by CD11chigh MCH-IIhigh and MCH-II+ CD11b+ F4/80+ expression, respectively. Resident monocytes were characterizedby the expression of CD11bint Ly6Cint Gr1−, inflammatory monocytes by the expression of CD11bint Ly6Chigh Ly6G− or CD11bint Gr1int and MDSC by the expression of CD11bhigh Ly6Cint Ly6G+ or CD11bhigh Gr1high. Inflammatory myeloid cells were considered tolerogenic when IDO expression was detected intracellularly. For Treg cells analysis cells were separated by the expression of CD4+ followed by gating on CD25+ FoxP3+. Finally, the antitumor specific response were caracterized by E7-specificIFN-γ+ producing CD8+ T cells. [file Data_Sheet_1.PDF]
